# Task shifting from general practitioners to nurses and the association with patient flow and resource use in out-of-hours primary care clinics: a descriptive register-based study

**DOI:** 10.1080/02813432.2026.2653004

**Published:** 2026-04-08

**Authors:** Katrine Bjørnshave Bomholt, Mette Amalie Nebsbjerg, Claus Høstrup Vestergaard, Anna Mygind, Viola Burau, Morten Bondo Christensen, Linda Huibers

**Affiliations:** ^a^ Research Unit for General Practice, Aarhus, Denmark; ^b^ Department of Public Health, Aarhus University, Aarhus, Denmark; ^c^ Department of Respiratory Diseases and Allergy, Aarhus University Hospital, Aarhus, Denmark; ^d^ Department of Gynaecology and Obstetrics, Aarhus University Hospital, Aarhus, Denmark

**Keywords:** Primary health care, after-hours care, task shifting, general practice, register study

## Abstract

**Background:**

This study aims to describe patterns of patient flow and resource use in out-of-hours (OOH-PC) clinics with high versus low levels of task shifting from general practitioners (GPs) to nurses.

**Methods:**

This descriptive register-based study used data from OOH-PC clinics in the Central Denmark Region. Clinics with high levels of task shifting had nurses independently managing pa-tients with minor conditions, whereas in clinics with low levels of task shifting nurses mainly per-formed preparatory tasks. Measures of patient flow included GP time per contact, initial and total waiting time, contact time with a health professional, and treatment time. Resource use measures in-cluded diagnostic tests, X-rays, injury-related procedures, prescriptions, hospital referrals, and fol-low-up contacts.

**Results:**

A total of 451,714 contacts were included. The proportion of contacts independently man-aged by nurses was 4% in clinics with low task shifting and 20% in clinics with high task shifting. Clinics with low task shifting had more GP time per contact (median 8 minutes [IQR 5–11] vs. 6 minutes [IQR 2–11]) and longer total waiting time (31 minutes [IQR 14–52] vs. 26 minutes [IQR 11–49]) than clinics with high task shifting. Median contact time (8 minutes) was similar across clin-ic types. Rates of diagnostic testing, X-ray referrals, prescriptions, and follow-up contacts were comparable.

**Conclusions:**

This study indicates that OOH-PC clinics with high levels of task shifting release more GP time and reduce waiting times while maintaining similar consultation lengths and overall resource use.

**Clinical trial number:**

Not applicable.

## Background

Regardless of the access point, patients requiring acute care must receive appropriate, timely, and high-quality care from health professionals at the most suitable healthcare setting [1–3]. In many Western countries, general practitioners (GPs) provide out-of-hours primary care (OOH-PC) through different organisational models, including large-scale GP cooperatives (GPCs) [2–4]. However, OOH-PC is facing increasing pressure due to an ageing population, increased prevalence of chronic diseases, and the transfer of tasks from hospitals to primary care [5–9]. This pressure may lead to reduced patient satisfaction, longer waiting times, and increased risk of delayed treatment and safety incidents [10–14]. Workforce shortage and declining motivation among GPs to take shifts further challenges the sustainability of these services [3,10,15].

Innovative workforce strategies are one way to address these challenges [16]. Task shifting, where specific responsibilities are reallocated from GPs to other health professionals such as nurses, is one potential approach [15,17–22]. In various healthcare settings, including daytime general practice, task shifting has been associated with safe care, high patient satisfaction and reduced GP workload [17,18,20,23–25].

Evidence from daytime general practice may not be directly transferable to OOH-PC because the context differs substantially [26]. OOH-PC focuses on urgent care and often involves unfamiliar teams working together under time pressure [27,28]. While task shifting to nurses has been widely implemented for telephone triage in OOH-PC [29–31], task shifting in clinic consultations remains less studies [26,28,32,33]. Therefore, we aim to describe patterns of patient flow and resource use in OOH-PC clinics with different levels of task shifting from GPs to nurses.

## Methods

### Design and participants

We conducted a descriptive register-based study using data on clinic consultations conducted at five OOH-PC clinics in the Central Denmark Region from 1 January 2018 until 31 December 2022. We included all clinic consultations from 5 pm to 10 pm on weekdays and from 8 am to 10 pm on weekends to ensure comparable data collection. On weekdays, telephone triage began at 4 pm, with patient inflow to clinics typically starting around 5 pm. On weekends, overnight telephone triage was available, but most clinics opened at 8 am for consultations. Most clinics closed at 11 pm, but nurse shifts often ended by 10 pm.

### Setting

The Danish healthcare sector is dominated by the tax-funded public sector, which provides free public healthcare to residents [34]. Patients presented with undifferentiated symptoms and acute health problems that can be assessed and managed in OOH-PC, similar to daytime general practice. General practice and OOH-PC acts as gatekeepers to secondary care, ensuring that specialist and hospital services are only provided when necessary [35]. During the study period, OOH-PC services were open on weekdays from 4 pm to 8 am and for 24 h during weekends and holidays [35]. The Central Denmark Region is the second-largest region in Denmark, with approximately 1.4 million residents and covering an area of 13,008 km^2^.

At the time of data collection, the OOH-PC service in the Central Denmark Region was provided by a large-scale GPC organisation with ten clinics varying in geography, size, staffing, facilities, opening hours, and level of collaboration with an ED [35]. Patients accessed the OOH-PC service by calling one regional telephone number. Danish GPs and GP trainees performed telephone triage and determined the appropriate level of care: advice or treatment by telephone (with/without video use), referral to an OOH-PC clinic consultation, home visit by a GP, or referral directly to a hospital [36]. At all OOH-PC clinics, a range of diagnostic tools were available, including C-reactive protein (CRP) test, rapid strep A test, blood glucose test, haemoglobin test, dipstick urinalysis (U-stick), pregnancy test, and electrocardiogram (ECG). Furthermore, referral to an in-house X-ray was possible.

Clinics provided care to patients in teams consisting of nurses and GPs. The GPs were required to take OOH shifts due to contractual obligations with the regional health services and rotated between telephone triage, clinic consultation and home visit shifts. The GPs were responsible for all treatment provided in the OOH-PC clinics and were paid a fee-for-service calculated through remuneration codes. The nurses working at the OOH-PC clinic were employed at an ED and received an hourly salary; they could have alternating shifts between the ED and the OOH-PC clinic.

### Intervention: task-shifting model

We utilised naturally occurring variation in the organisation and use of task shifting from GPs to nurses across OOH-PC clinics in the Central Denmark Region, informed by findings from a previous explorative case study [37].

Clinics were purposefully selected and categorised by the research group into high and low levels of task shifting based on observed practices and the role of nurses in patient management (Table 1).

**Table 1.** Characteristics of OOH-PC clinics categorised by level of task shifting.

Characteristic	Low level of task shifting (*n* = 3)	High level of task shifting (*n* = 2)
*Context*	Located within hospitals that include an ED; clinics situated in rural areas.	Co-located with an acute care clinic functioning as an ED satellite for trauma; clinics situated in urban areas.
*Team*	One nurse worked with one to four GPs. Nurse moved flexibly between the ED and the OOH-PC clinic, depending on the workload.	One nurse worked consistently with one GP. Same nurse managed the acute care clinic and the OOH-PC clinic, receiving telephone supervision from an ED doctor, including discussion of X-ray results and completion of treatment.
*Nurse experience and training*	The nurses had various levels of experience and training.	Nurses always received additional training enabled independent management of injury-related conditions including removal of foreign bodies from the eye, ear, or nose, application of immobilising bandages, management of minor fractures or joint dislocations, and treatment of larger wounds. They could refer patients for X-ray examinations of suspected bone injuries. They did not have prescribing authority. Most nurses had extensive clinical experience from ED and OOH-PC.

ED: emergency department; GP: general practitioner; *n*: number; OOH-PC: Out-of-hours primary care.

- Clinics with high levels of task shifting were characterised by nurses independently assessing and managing a substantial proportion of patients presenting with minor conditions, such as wounds and minor musculoskeletal injuries and completed consultations with GP support available when needed. In these clinics, nurses also frequently initiated diagnostic testing upon patient arrival.
- Clinics with low levels of task shifting were characterised by nurse involvement primarily limited to preparatory and supportive tasks, while clinical assessment, decision-making, and treatment were conducted by GPs.

### Outcome measures

We defined outcome measures for patient flow and resource use based on existing literature and observations from the explorative case study [41].

**Figure 1.**
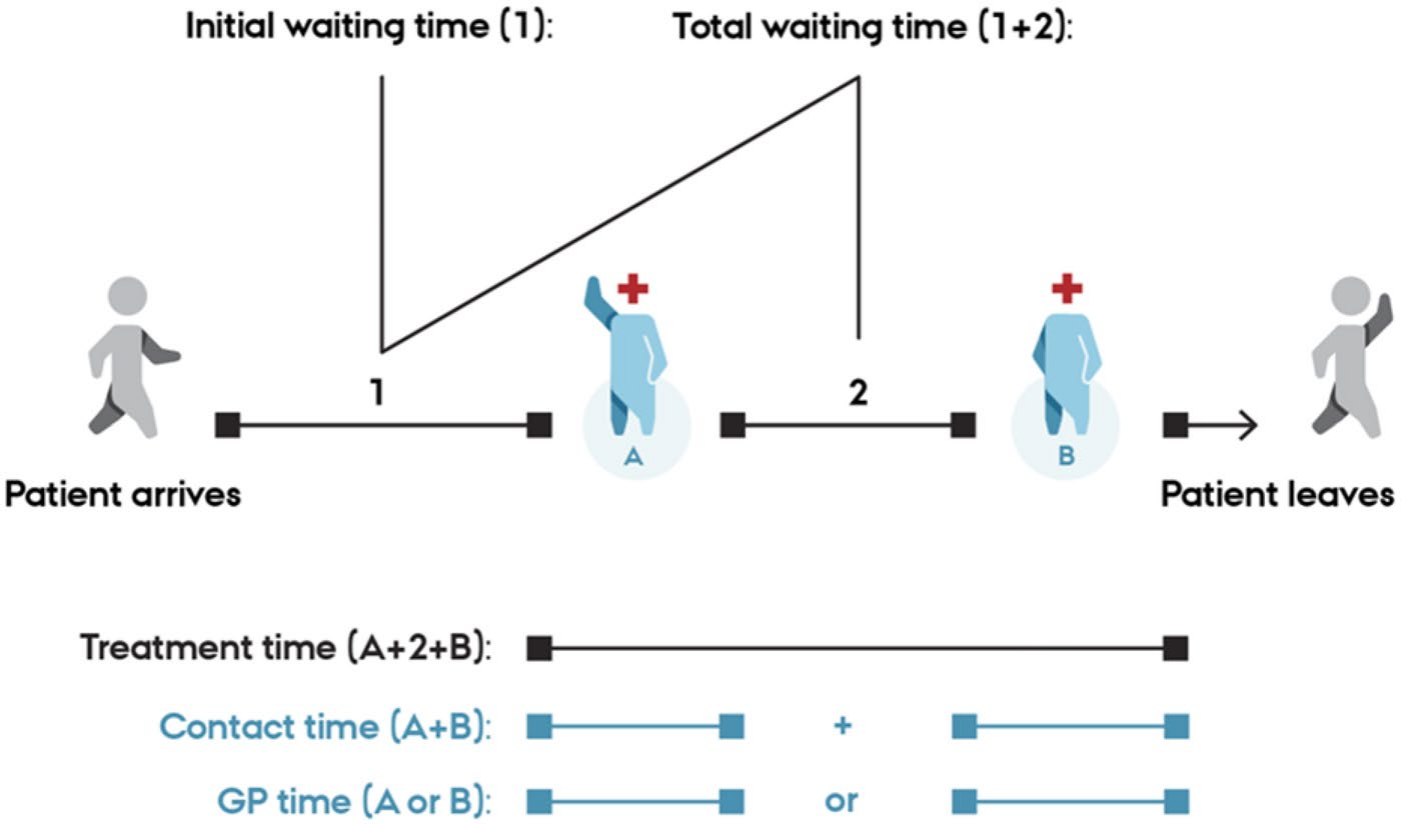
Definition of patient flow outcome measures. GP: general practitioner. A and B denote the contact time with a health professional (GP or nurse).

Five outcome measures related to patient flow (Figure 1):

- GP time: face-to-face time spent with a patient and time used for documentation
- Initial waiting time: time from arrival at clinic to first contact with a GP or nurse
- Total waiting time: time spent in the waiting room, including initial waiting time and any waiting time between encounters with a GP and/or nurse
- Contact time: face-to-face contact time spent between patient and GP and/or nurse
- Treatment time: time spent from start to end of face-to-face contact with health professional(s), including waiting time between meetings with another or the same health professional(s).

Six outcome measures related to resource use:

- Diagnostic tests: CRP, strep A, blood glucose, haemoglobin, pregnancy (hCG) tests, U-stick, ECG
- X-ray of bones and thorax
- Injury-related procedures: removal of foreign objects in eye/ear/nose, immobilising bandage, treatment of bone fractures, joint slips, and large wounds
- Prescription of medication: pain, antibiotics, and other
- Referral to hospital department
- Follow-up contact in daytime general practice or OOH-PC within seven days or hospital admission within one day.

### Data collection

We used data from the OOH-PC electronic registration system, which provided information on the date and time of contact, clinic, diagnostic tests, X-rays, procedures, prescriptions, and referrals. To investigate follow-up contacts, we linked data from the OOH-PC registration system to two Danish national registers using the patient’s unique personal identification number [38]. The Danish National Health Service Register provided information on the date and type of contacts to daytime general practice and the OOH-PC service [39], and the Danish National Patient Registry provided information on the date of contact with the hospital and diagnosis codes [40]. Data on the socioeconomic characteristics of the patient (i.e. sex, age, cohabitation, educational level, ethnicity, income, urbanisation, and employment status) was obtained from Statistics Denmark [41].

### Data management

We constructed an “independent nurse contact” variable, defined by GP consultation length ≤ 60 s, as we assumed that no face-to-face time with a GP could be managed in such a short period. Instead, such short GP contacts were assumed to indicate GPs reading nurse notes in the patient records and coding for remuneration. As this administrative task could be done by the GP, potentially long after the patient has left the clinic, such short period of time was ignored when calculating treatment times and waiting time. Thus, all GP time with a consultation length ≤ 60 s was set at zero seconds.

Prescriptions were categorised according to the Anatomical Therapeutic Chemical (ATC) classification system into three subgroups: antibiotics, painkillers, and others (all other types of medication, e.g. medicines for asthma, eczema, and nausea). Using the diagnosis codes in hospital charts, we defined level of comorbidity based on the number of chronic diagnoses listed in the Charlson Comorbidity Index [42]. Except for age, sex, and comorbidity, all covariates were reported at household level. For example, educational level was determined by the household member with the longest education. Hence, it was possible to estimate children’s socioeconomic status through information about their parents.

### Data analysis

The study population was described and presented as numbers (*n*) and percentages (%). Patient flow outcomes were summarised by their mean, median, and interquartile range (IQR). Resource use outcomes were presented as numbers (*n*) and percentages (%).

All calculations and data management were carried out using R, version 4.1.1. Results were reported in accordance with the Strengthening the Reporting of Observational Studies in Epidemiology (STROBE) statement [43].

## Results

### Descriptive data

Overall, 451,714 patient contacts were included: 81% from clinics with low levels of task shifting and 19% from clinics with high levels (Supplementary Table 1). Minor differences were observed between patient groups: clinics with low task shifting had a lower proportion of native Danish patients and a higher proportion of patients in the lowest income quintile.

### Distribution of contacts

In low task shifting clinics, 83% of contacts were managed by only a GP, 13% by a GP and a nurse, and 4% by a nurse independently. In high task shifting clinics, 60% of contacts were managed by only a GP, 20% by a GP and a nurse, and 20% by a nurse independently.

### GP time

**Figure 2.**
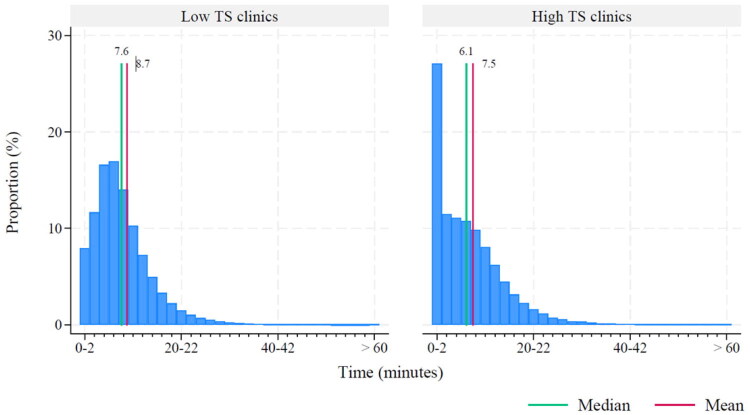
Time distribution for GPs (in %) in OOH-PC clinics with low and high task shifting. Time: face-to-face time spent by a GP on a patient, including documentation; TS: task shifting.

The median GP time spent per patient was 8 min [IQR 5–11 min] in low task shifting clinics vs. 6 min [IQR 2–11 min] in high task shifting clinics (Figure 2).

### Waiting time

The median *initial* waiting time was 27 min [IQR 11–48 min] in low task shifting clinics vs. 19 min [IQR 7–41 min] in high task shifting clinics. The median *total* waiting time was 31 min [IQR 14–52 min] in low task shifting clinics vs. 26 min [IQR 11–49 min] in high task shifting clinics.

### Contact and treatment time

The median contact time with a health professional (GP and/or nurse) was 8 min in both types of clinics (low task shifting clinics IQR: 5–12 min, high task shifting clinics IQR: 4–13 min). The median treatment time was 9 min for both types of clinics (low task shifting clinics IQR: 5–14 min, high task shifting clinics IQR: 5–17 min).

### Resource use

Clinics with low levels of task shifting had similar rates of diagnostic tests (both 40%), X-ray referrals (7% vs. 6%), and prescriptions (25% vs. 23%) compared to high task shifting clinics. Also, clinics with low levels of task shifting had similar follow-up contacts (daytime general practice contacts: 49% vs. 50%, OOH-PC contacts: both 10%, and unplanned hospital contacts: both 2%). In contrast, procedures performed were slightly lower (19% vs. 23%), and referrals to hospital departments were higher (14% vs. 8%), in low task shifting clinics compared to high task shifting clinics (Table 2).

**Table 2.** Distribution of resource use in OOH-PC clinics, stratified for level of task shifting.

	Low TS *n* (%)	High TS *n* (%)
*Diagnostic tests (total)*	145,958 (40)	34,540 (40)
U-stick	33,193 (9)	8246 (10)
Haemoglobin	4501 (1)	904 (1)
Strep A	15,293 (4)	3427(4)
CRP	74,186 (20)	18,060 (21)
Blood glucose	2575 (1)	626 (1)
ECG	11,426 (3)	2224 (3)
HCG	4787 (1)	1053 (1)
*Procedures (total)*	69,595 (19)	19,902 (23)
Removal of foreign body (eye/ear/nose)	8730 (2)	2456 (3)
Immobilising bandage	14,862 (4)	5518 (6)
Bone fracture and joint slip	6758 (2)	1199 (1)
Large wound	39,245 (11)	10,729 (12)
*X-ray*	26,162 (7)	4842 (6)
*Prescription (total)*	90,692 (25)	19,896 (23)
Painkillers	11,681 (3)	2238 (3)
Antibiotics	61,281 (17)	14,091 (16)
Other medications	17,730 (5)	3567 (4)
*Referrals*	(14)	(8)
*Follow-up (total)*	221,595 (61)	52,648 (61)
Daytime GP within 7 days	178,304 (49)	42,773 (50)
OOH-PC within 7 days	37,595 (10)	8350 (10)
Unplanned hospital contacts within 24 h	5693 (2)	1525 (2)

CRP: infection number; ECG: electrocardiogram; GP: general practice; hCG: haemoglobin; pregnancy tests; Strep A: rapid strep A; TS: task shifting; U-stick: dipstick urinalysis.

## Discussion

### Summary of results

This study described patterns of patient flow and resource use in OOH-PC clinics with different levels of task shifting from GPs to nurses. Clinics with high levels of task shifting had markedly greater proportions of contacts managed by nurses independently, and these GPs generally spent less time per contact. Clinics with high levels of task shifting also had shorter initial and total waiting times. Consultation length and treatment time were similar for both clinic types, as were patterns of diagnostic testing, X-ray use, prescribing, and follow-up contacts. However, clinics with high task shifting conducted a higher proportion of injury-related procedures and had lower rates of referrals to hospital departments.

### Strengths and limitations

This study was based on a large dataset comprising GP remuneration codes. The economic incentive for GPs to register all provided services contributed to the completeness of the dataset, though validity has not yet been studied [39]. Our design enabled examination of real-world differences in patient flow and resource use between clinics with varying levels of task shifting, as we purposively selected clinics with both high and low levels of task shifting. Selecting more than one clinic in each group ensured diversity in organisational structures, team dynamics, and local implementation of task shifting, thereby enhancing the generalisability of our findings. Furthermore, hypothesis generation was enhanced by the descriptive nature of our study and the knowledge obtained in the explorative case study [41].

Our study also had some limitations. First, tasks performed by nurses are not systematically registered, so we could not compare tasks performed by nurses to those performed by GPs. Second, we had no information on the reason for encounter, as this is not systematically registered in OOH-PC contacts. Triage to a consultation at an OOH-PC clinic is based on travel distance combined with level of urgency, complexity, and expected need for care, as facilities at the OOH-PC clinic location can vary. Thus, patients visiting OOH-PC clinics co-located with an ED or a hospital could have a different diagnostic scope. Consequently, our findings might have been influenced by confounding by indication, as the clinics with high level of task shifting were not co-located with an ED. Therefore, these clinics may have had a higher proportion of patients presenting with minor health problems that could be handled by nurses alone and required fewer referrals to the ED or hospital. Third, we followed each patient for seven days to record follow-up contacts in OOH-PC and daytime general practice, as previously described in the literature [44]. This large follow-up period may have overestimated the number of follow-up contacts, as we could not exclude follow-up contacts that were unrelated to the index contacts at the OOH-PC due to the lack of reason for encounter. However, any overestimation would be independent of the level of task shifting in the clinics. Finally, patient characteristics varied between clinics with low versus high levels of task shifting. We assume that most differences were related to the location of the clinics (urban versus rural).

Several factors must be considered when generalising the results from this study. First, the study period included the COVID-19 pandemic period, which likely changed contact patterns, patient flow, and resource use in OOH-PC [44–46]. This could have introduced variations in care delivery that do not reflect normal operating conditions, which may limit the external validity and generalisability to the post-pandemic period. To mitigate these effects, we included a relatively long period before and after the COVID-19 pandemic. Moreover, we assumed that pandemic-related differences in operating conditions would have affected the included clinics similarly. Second, the study was conducted in the Danish OOH-PC setting with large-scale GP cooperatives [2]. The findings may not directly apply to other healthcare systems, as these organisations can vary between and within countries [1,3].

### Comparison with existing literature

In relation to patient flow, our findings indicate that high levels of task shifting may release GP time in OOH-PC clinics. This is consistent with existing evidence showing that nurses are well-suited to manage patients with less urgent and non-complex conditions, thereby allowing GPs to concentrate on patients requiring more complex assessment [28,47]. Previous studies have also shown that nurses provide care of comparable quality to that of GPs for conditions such as minor trauma and skin complaints [26], and that a substantial proportion of OOH-PC contacts fall within the competences of nurses with additional training [5,28,48]. Our results align with these findings, as a notable proportion of contacts in clinics with high task shifting were completed by nurses independently, suggesting that trained nurses already absorb a clinically meaningful proportion of the OOH-PC case load.

While OOH-PC research has reported lower productivity among nurses, partly due to longer consultation times [27], we found that clinics with high task shifting did not differ from those with low task shifting in terms of median contact time and treatment times. This divergence may reflect the organisational setup. Much of the existing literature compares individual consultations conducted by nurses and GPs. In our study, clinics with high task shifting level operated through coordinated, interprofessional workflows. Nurses handled diagnostic tests and managed injuries before GP involvement, enabling GPs to proceed directly with clinical decision-making [37]. These findings are consistent with literature highlighting that interprofessional collaboration improves efficiency by integrating complementary skills and allocating tasks in ways that advance shared clinical objectives [47,49–52].

### Implications for practice and future research

A high level of task shifting in OOH-PC clinics may help address the challenges of high workload and GP shortage by releasing GP time. However, further research is needed to investigate the effect of task shifting on GP workload and patient outcomes. Importantly, future studies should take the diagnostic scope into consideration to avoid confounding by indication. A randomised controlled trial, with a random distribution of patients or health professionals across clinics, would provide robust evidence on the impact of task shifting. Given the workforce shortage, exploring the involvement of healthcare professionals with varying backgrounds in task shifting models, such as paramedics, nurses without additional training, and medical students, could have potential. Future research should examine the feasibility, safety, and acceptability of involving these professionals in OOH-PC to ensure that the quality of care is maintained while addressing workforce challenges.

## Conclusion

This study indicates that OOH-PC clinics with high levels of task shifting can release GP time and shorten waiting times while maintaining comparable consultation lengths and similar use of diagnostic tests, medication prescribing, and follow-up care. The higher frequency of procedures and the lower rate of hospital referrals in these clinics suggest that nurses undertake a broad set of clinical tasks, thereby reducing the need for direct GP involvement and downstream hospital care. Further research should examine how different task shifting models influence clinical outcomes, patient safety, and patient experience, as such new knowledge could inform future workforce planning in OOH-PC.

## Supplementary Material

Supplement.docx

## Data Availability

The data are not publicly available due to confidentiality and ethical restrictions, but anonymised data can be obtained from the corresponding author upon reasonable request.
