# Supplementary material for: Task shifting from general practitioners to nurses and the association with patient flow and resource use in out-of-hours primary care clinics: a descriptive register-based study
Source: Scand J Prim Health Care. 2026 Apr 8;44(1):2653004. doi: 10.1080/02813432.2026.2653004 (PMC13063318; doi:10.1080/02813432.2026.2653004)
Supplement: Supplement.docx [file IPRI_A_2653004_SM0212.docx]

**Supplement**

**Table 1:** Distribution of patient and contact characteristics at OOH-PC clinics, stratified for level of task shifting

|  | **Low TS**  (n=365,419)  n (%) | | **High TS**  (n=86,295)  n (%) | **Total**  (n=451,714)  n (%) |
| --- | --- | --- | --- | --- |
| **Age (years)** | | | | |
| 0-4 | 24,658 (7) | | 5,521 (6) | 30,179 (7) |
| 5-10 | 45,274 (12) | | 10,533 (12) | 55,807 (12) |
| 11-20 | 47,593 (13) | | 12,793 (15) | 60,386 (13) |
| 21-40 | 117,420 (32) | | 22,188 (26) | 139,608 (31) |
| 41-60 | 72,785 (20) | | 18,915 (22) | 91,700 (20) |
| 61-80 | 47,349 (13) | | 13,284 (15) | 60,633 (13) |
| +81 | 10,340 (3) | | 3,061 (4) | 13,401 (3) |
| **Sex** | | | | |
| Female | 189,957 (52) | | 43,992 (51) | 233,949 (52) |
| Male | 175,462 (48) | | 42,303 (49) | 217,765 (48) |
| **Comorbidities (n)** | | | | |
| None | 319,784 (88) | | 74,887 (87) | 394,671 (87) |
| 1 | 36,204 (10) | | 8,963 (10) | 45,167 (10) |
| 2 | 7,077 (2) | | 1,831 (2) | 8,908 (2) |
| >2 | 2,354 (1) | | 614 (1) | 2,968 (1) |
| **Cohabitation status** | | | | |
| Single | 118,901 (33) | | 23,329 (27) | 142,230 (31) |
| Cohabiting | 80,489 (22) | | 18,381 (21) | 98,870 (22) |
| Married | 160,298 (44) | | 43,309 (50) | 203,607 (45) |
| Missing | 5,731 (2) | | 1,276 (1) | 7,007 (2) |
| **Education (years)** | | | | |
| >15 | 86,927 (24) | | 19,595 (23) | 106,522 (24) |
| 10-15 | 149,440 (41) | | 36,940 (43) | 186,380 (41) |
| <10 | 122,972 (34) | | 28,410 (33) | 151,382 (34) |
| Missing | 6,080 (2) | | 1,350 (2) | 7,430 (2) |
| **Ethnicity** | | | | |
| Native | 309,176 (85) | | 78,643 (91) | 387,819 (86) |
| Western | 11,171 (3) | | 1,891 (2) | 13,062 (3) |
| Non-western | 39,341 (11) | | 4,485 (5) | 43,826 (10) |
| Missing | 5,731 (2) | | 1,276 (1) | 7,007 (2) |
| **Income (quintile)** | | | | |
| 1^st^ (lowest) | 84,153 (23) | | 14,191 (16) | 98,344 (22) |
| 2^nd^ | 72,747 (20) | | 16,439 (19) | 89,186 (20) |
| 3^rd^ | 70,816 (19) | | 18,655 (22) | 89,471 (20) |
| 4^th^ | 68,341 (19) | | 19,133 (22) | 87,474 (19) |
| 5^th^ (highest) | 63,631 (17) | | 16,601 (19) | 80,232 (18) |
| Missing | 5,731 (2) | | 1,276 (1) | 7,007 (2) |
| **Urbanisation** | | | | |
| > 100,000 | 134,645 (37) | | 2,990 (3) | 137,635 (30) |
| 20,000-100,000 | 73,646 (20) | | 27,443 (32) | 101,089 (22) |
| 1,000-20,000 | 84,483 (23) | | 31,919 (37) | 116,402 (26) |
| < 1,000 | 64,425 (18) | | 22,267 (26) | 86,692 (19) |
| Unknown | 2,391 (1) |  | 383 (0) | 2,774 (1) |
| Missing | 5,829 (2) |  | 1,293 (1) | 7,122 (2) |

^N: number, TS: task shifting^
